# An individualized physical function improvement program for frailty elderly using goal attainment scaling (GAS) as evaluation method: protocol for a randomized controlled trial

**DOI:** 10.1186/s12877-025-06203-1

**Published:** 2025-07-15

**Authors:** Yu Kang, Zi-Cheng Qi, Meng-Tian Guo, Xiao-Juan Wang

**Affiliations:** Department of General Practice, Beijing Chaoyang Hospital, Capital Medical University, 8 Gongren Tiyuchang Nanlu, Chaoyang District, Beijing, 100020 China

**Keywords:** Frailty, Goal attainment scaling, Physical function, Study protocol, Geriatric

## Abstract

**Background:**

Frailty is the deterioration of physiological functions due to aging, linked to negative outcomes. Frailty, being a reversible and dynamic state, calls for individualized and comprehensive management. Goal Attainment Scaling (GAS) is a personalized method for establishing and assessing progress towards individual goals, suitable for older adults with complex requirements. We focus on attaining personalized intervention goals for elderly patients with frailty, correcting frailty manifestations, maintaining enhanced functional capacity, and cultivating sustainable health awareness, with the goal of improving overall quality of life.

**Methods:**

We conducted a randomized clinical trial of an individualized intervention program using GAS as goal setting and evaluation method designed to improve physical functional in frailty elderly. A total of 160 individuals aged ≥ 60 years, who fulfill the Fried scale of frailty will be recruited from Beijing Chaoyang Hospital, Capital Medical University. All participants set personalized goals through GAS. Patients in the intervention group receive individualized interventions, implement tailored measures based on personalized goals, attain personalized intervention goals to improve or reverse the frailty state. The participants will be followed up for 3 months and 12 months.

**Discussion:**

The advantage of the present study is the method of intervention. Personalized interventions will implement based on patient-specific needs through individualized goal-setting formulated according to the GAS. If a positive consequence could be obtained, this study’s findings will offer essential information for managing frailty in older adults, applicable in regular clinical settings.

**Ethics and trial registration:**

The trials have received ethics approval from the Institutional Review Board for Human Studies of Beijing Chaoyang Hospital, Beijing, China. The clinical study is supported by the National Health Commission of the People’s Republic of China (2024YB53). We declare that the funding agency has independently reviewed the protocol as part of the process of awarding funding. The trial is registered as ClinicalTrials.gov Identifier: NCT06919575 (Registration Date: 2025-04-02).

**Supplementary Information:**

The online version contains supplementary material available at 10.1186/s12877-025-06203-1.

## Background

The world’s population is getting older as average life expectancy rises, with 21.3% expected to be 60 or older by the year 2050. Healthcare systems are facing a growing challenge with higher demand because of the aging population, with frailty among older adults is becoming more common worldwide. Literature reported that the prevalence of frailty and pre-frailty in older adults was 17.4% and 52.6%, respectively [1]. Efforts to reduce the burden of frailty could have substantial public health consequences [2, 3].

Increasing attention is being paid to the growing prevalence of frailty in older adults. Frailty involves a notable reduction in the functional reserve of various organ systems, making individuals more susceptible to stressors, resulting in a greater risk of adverse health outcomes, including falls, disability hospitalization and mortality among older adults [4–10]. Thus, enhancing the functional abilities of frail older adults and potentially reversing their frailty is of great clinical significance.

Frailty is described as a dynamic transition, that can be improved or reversed. This phenomenon was confirmed by several trials conducted on different types of individuals, and the cost associated to them avoidable, thus preventing imminent future society demands [11–17]. Studies have indicated that frailty transitions are affected by factors such as older age, previous health issues, physical activity and mobility levels, socio-economic and clinical conditions, and vitamin D levels [18–25]. Addressing these changeable factors could improve the functional status of frail elderly people.

Several studies showed that multi-domain interventions of frailty are effective in improving the frail status of individuals [26–33]. Notably, targeted therapies are highly advised over non-specific treatments in precision medicine. However, evidence remains limited regarding personalized frailty intervention protocols tailored to individual clinical profiles.

Frailty people frequently experience a multitude and diversity of health problems that cause functional limitations. Since the impact of these health problems varies from person to person, the priorities and goals of treatment also differ for each older individual. Standardized assessment tools often fail to adequately measure intervention effects across this heterogeneous population. The diversity of patients, diseases and personal goals makes it important to be able to evaluate the attainment of all relevant goals per patient. Given the heterogeneity in individual requirements, personalized intervention strategies should be formulated based on comprehensive geriatric assessments.

Goal setting is a key component of person-centered, individualized interventions. Goal Attainment Scaling (GAS) is a validated outcome measurement tool that facilitates the establishment and quantitative assessment of personalized treatment goals [34–40]. This method is particularly suitable for older adults with multiple chronic conditions and complex needs, as it enables tailored goal setting and structured evaluation of progress. A key feature of GAS is its ability to individualize treatment goals while enabling standardized progress tracking, yielding a quantifiable and comparable score across individuals. As a personalized outcome measure, GAS supports the assessment of multiple patient-specific objectives, including those in tailored interventions. By systematically integrating functional assessments and individualized needs, GAS enhances precision in tracking progress and refining intervention strategies.

We conduct a randomized clinical trial of an individualized intervention program using Goal Attainment Scaling (GAS) as evaluation method designed to Improve physical functional in frailty elderly. Our primary aim focused on attaining personalized intervention goals for elderly patients with frailty, correcting frailty manifestations, maintaining enhanced functional capacity, and cultivating sustainable health awareness, with the goal of improving overall quality of life.

## Methods

### Study design

**Fig. 1:**
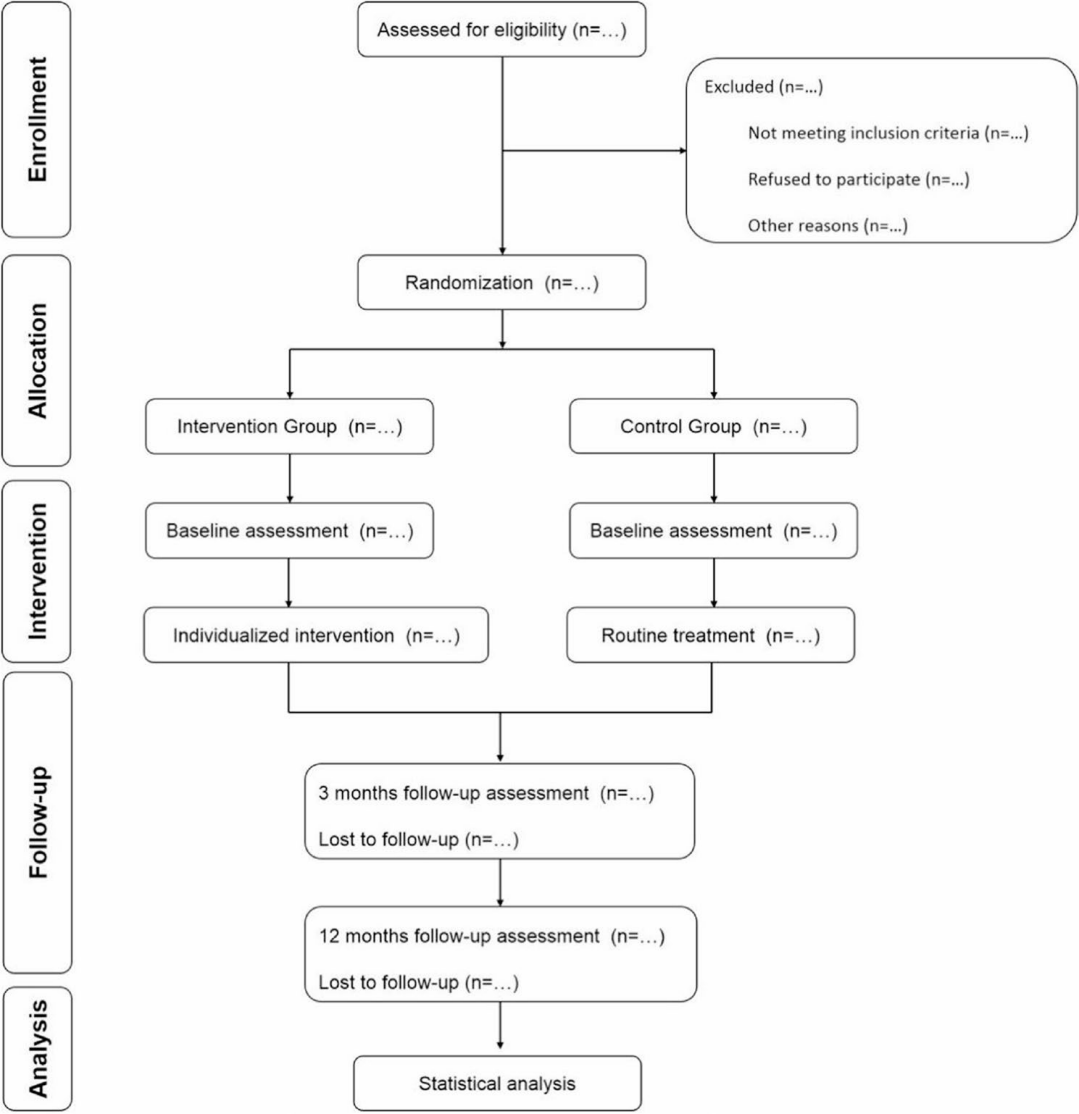
Schematic representation of the randomized controlled trial

This randomized parallel controlled trial included a 3-month and 12-month follow-up period. Figure 1 shows the flow diagram of the progress through the phases of this parallel randomized trial of two groups. Participants were recruited from a large general hospital and two community health centers within a medical alliance in Chaoyang District, Beijing. The study protocol received approval from the Institutional Review Board for Human Studies of Beijing Chaoyang Hospital. Funding was provided by the National Health Commission of the People’s Republic of China (Grant No. 2024YB53). The trial is registered at ClinicalTrials.gov NCT06919575 (Registration Date: 2025-04-02).

### Recruitment

The recruitment process comprises two stages:

- Screening Phase: Potential participants aged ≥ 60 years will be screened for frailty at a large general hospital and two community health service centers within a medical alliance. A brief assessment will evaluate inclusion/exclusion criteria, with outpatient clinics serving as the primary recruitment source.
- Enrollment Phase: Eligible individuals expressing interest will receive detailed study information. Written informed consent will be obtained prior to participation. Recruitment will continue until the predetermined sample size for statistical analysis is achieved.

### Measures of frailty

Frailty will be identified using Fried scale according to its five criteria [41–43]:

1. Weight loss: defined as unintentional weight loss ≥ 4.5 kg or ≥ 5% of body weight in the last year.
2. Exhaustion: was measured by self-report using two questions: (‘How many days during the last week have you felt that anything you did was a big effort?’ and ‘How many times during the last week have you felt that you could not keep on doing things?’). The criterion was met when participant answered self-reported fatigue ≥ 3 days in the last week.
3. Weakness: based on grip strength, stratified by gender and body mass index (BMI) quartiles. For men, BMI ≤ 24 kg/m^2^, grip strength ≤ 29 kg; BMI 24.1–26 kg/m^2^, grip strength ≤ 30 kg; BMI 26.1–28 kg/m^2^, grip strength ≤ 30 kg; BMI >28 kg/m^2^, grip strength ≤ 32 kg. For women, BMI ≤ 23 kg/m^2^, grip strength ≤ 17 kg; BMI 23.1–26 kg/m^2^, grip strength ≤ 17.3 kg; BMI 26.1–29 kg/m^2^, grip strength ≤ 18 kg; BMI > 29 kg/m^2^, grip strength ≤ 21 kg. In this study, we will measure the grip strength of the dominant hand twice to obtain the average.
4. Slowness: was defined using the 6 m walking test at their usual pace, ≤ 1.0 m/s is considered abnormal. Best time of two performances was chosen.
5. Low physical activity: defined as energy expenditure of physical activity per week <383 kcal for men or <270 kcal for women.

The presence of ≥ 3 criteria is defined as frailty.

### Inclusion and exclusion criteria

The inclusion criteria were the following: (1) ≥ 60-years old; (2) recognized as frailty by Fried scale.

The exclusion criteria were the following: (1) considered with life expectancy <1 year such as advanced cancer patients; (2) difficult to communicate with such as patients with severe cognitive impairment (Mini Mental State Examination score ≤ 17); (3) severe hearing discord.

### Randomization

Once a patient fulfills the criteria, after informed consent, participants will be randomly assigned to either the Usual care group or the Intervention group based on random number at a 1:1 ratio generated in a blinded manner by the computerized randomization system. The information of random group allocation will be maintained in a sealed envelope opened only by a trial manager who is not involved in the recruitment, assessment, or intervention to assign the treatment. The control group will receive routine diagnosis and treatment, while the intervention group will receive individualized interventions.

### Justification of sample size

The sample size was calculated using a two-proportion comparison formula for a 1:1 randomized controlled trial, with an α of 0.05, 80% power, and an expected 20% absolute difference in frailty reversal rates between groups, based on prior intervention effect sizes. Initial calculations indicated 64 participants per group. Accounting for a 20% attrition rate, the adjusted sample size was 80 per group, resulting in a total of 160 participants. (http://powerandsamplesize.com/Calculators/)

## Intervention

First, a comprehensive assessment will first identify the underlying etiologies of frailty (e.g., physical inactivity, malnutrition, or psychological factors) to guide targeted interventions. Individualized treatment goals will be established using Goal Attainment Scaling (GAS), with subsequent implementation of personalized intervention plans. For participants presenting with ≥ 2 concurrent issues, multidisciplinary specialists will coordinate simultaneous management.

Participants identified with malnutrition or mood disorders will undergo standardized nutritional assessment (e.g., Mini Nutritional Assessment) and psychological evaluation (e.g., Geriatric Depression Scale). Evidence-based nutritional supplementation and/or pharmacotherapy will be initiated according to established clinical guidelines when indicated.

The patients of the intervention group with poor physical function would receive exercise prescription tailored to the subjects’ physical ability from a trained specialist. For Participants requiring exercise-based interventions, a moderate and gradually increasing intensity exercise regimen home-based, three times a week will be administer under telehealth-based monitoring. A standardized protocol exercise intervention comprises a 20-30-minute session with progressive phases, over a 12-week duration: 5-minute dynamic stretching warm-up, 10-minute moderate-intensity cardiovascular training, 5-minute progressive resistance exercises, 5-minute proprioceptive neuromuscular facilitation, and 10-minute isometric stance maintenance. Certified exercise specialists will individualize each prescription by modifying intensity, duration, and modality based on comprehensive geriatric assessment, while maintaining core therapeutic components to ensure safety, efficacy, and sustainable adherence.

### Formulation of intervention objectives and assessment of intervention efficacy

Intervention objectives and goal attainment will be evaluated using Goal Attainment Scaling (GAS), a standardized individualized methodology for establishing and assessing progress toward personalized objectives. The GAS process involves: (1) goal identification; (2) baseline status determination; (3) specification of outcome levels on a five-point ordinal scale (−2 to + 2); (4) goal weighting; and (5) follow-up scoring by comparing achieved outcomes with predefined attainment levels. This structured approach enables quantitative measurement of intervention efficacy across diverse clinical targets.

The research team formulated an initial pool of potential goals derived from the International Classification of Functioning, Disability, and Health (ICF) framework. Following rigorous focus group evaluations assessing goal relevance, clinical significance, and practical applicability, 25 individualized goal indicators were finalized. These indicators encompassed five key domains: body functions, mental functions, sensory functions, domestic life, and cardiopulmonary function, each tailored to meet participants’ specific needs and preferences. The complete set of 25 individualized goals is detailed in Table 1.

**Table 1:** Goals pool for individualized interventions on frailty elderly

Domain	Area	Category
Body functions	1.1 Mental functions	1.1.1 Orientation functions
		1.1.2 Energy and drive functions
		1.1.3 Sleep functions
		1.1.4 Attention functions
		1.1.5 Memory functions
		1.1.6 Emotional functions
	1.2 Sensory functions and pain	1.2.1 Proprioceptive function
		1.2.2 Sensation of pain
	1.3 Organic functions	1.3.1 Voice functions
		1.3.2 Heart functions
		1.3.3 Blood vessel functions
		1.3.4 Blood pressure functions
		1.3.5 Hematological system functions
		1.3.6 Respiration functions
		1.3.7 Ingestion functions
		1.3.8 Defecation functions
		1.3.9 Weight maintenance functions
		1.3.10 Water, mineral and electrolyte balance functions
Activity	2.1 Mobility	2.1.1 Exercise tolerance functions
		2.1.2 Mobility of joint functions
		2.1.3 Muscle power functions
		2.1.4 Control of voluntary movement functions
	2.2 Sensations of Mobility	2.2.1 Sensations related to muscles and movement functions
		2.2.2 Sensations associated with cardiovascular and respiratory functions
Environmental factors	3.1 Support and relationships	3.1.1 Family life functions

Through a structured collaborative process involving physicians, participants, and caregivers, each individual will select three personalized goals. Under the research team’s guidance, participants will actively engage in both goal-setting and baseline evaluations, assessing each goal’s importance and anticipated difficulty on a 0–3 scale. These ratings will be multiplied to generate a weighted goal score. Outcome measurement will employ a 5-point scale (−2 to + 2) where − 2 indicates much worse than expected, −1 somewhat worse, 0 expected achievement, + 1 somewhat better, and + 2 much better than expected outcomes. This standardized approach ensures systematic evaluation while maintaining individualization of therapeutic objectives.

Goal Attainment Scaling (GAS) scores were calculated using a standardized formula and evaluated at baseline, 3-month, and 12-month follow-ups. An aggregated T score with a mean of 50 and an SD of 10 was obtained. T is the composite score, wi is the weight assigned to the ith goal, xi is the numerical value (− 2 to + 2) of the attainment level of the ith goal. The extent to which goals are achieved is standardised into a T score by the formula:

$$\:GAS\:score=\:50\:+\:\:\:\:\frac{[10\sum\:\left(\text{w}\text{i}\times\:\text{x}\text{i}\right)]}{\surd\:[0.7\sum\:{wi}^{2}+{0.3\left(\sum\:wi\right)}^{2}]}$$

### Outcomes

The primary outcome measure was Goal Attainment Scaling (GAS) score, which quantitatively assessed individualized goal achievement. The secondary outcome is the reversal rate of frailty, defined as a reversion from frailty to pre-frailty or non-frailty.

### Statistical analysis

Outcome measures will be reported descriptively with 95% CIs. The GAS score and the reversal rate of frailty will be estimated between the intervention group and the control group. Responsiveness to the combined resistance band exercise and nutritional support intervention may exhibit significant variation across different ages within our target population of adults aged ≥ 60 years. To address this and avoid overgeneralization, we plan to conduct pre-specified subgroup analyses based on the following age brackets: 60–69 years, 70–79 years, and ≥ 80 years. These analyses will explore potential differences in intervention effectiveness thereby providing a more nuanced understanding of outcomes across the older adult spectrum. SPSS software (V.25, IBM Corp) will be used for statistical analysis.

### Adverse events

Potential adverse events (AEs) may include musculoskeletal pain, falls, nocturnal leg cramps and orthostatic hypotension. We will meticulously document all AEs and manage them appropriately. Participants who experience AEs will receive timely clinical assessment and appropriate medical care. All AEs will be discussed with the chief investigator who will assess seriousness and causality. AEs will also be reported to the study sponsor. Furthermore, all Suspected Unexpected Serious Adverse Reactions (SUSARs) will be reported to the ethics committee. SUSARs are defined as any episode during the study period that requires inpatient hospitalization, results in persistent/significant disability, is life threatening or results in death. The trial will be stopped prematurely if three or more SUSARs occur.

### Organization of the trial

The trial is structured with two operational units: a working group overseeing study design and a data collection group managing participant interactions. To optimize protocol compliance, each participant will be assigned a dedicated study staff member who will coordinate data collection visits, address study-related inquiries, and provide ongoing support. Regular meetings will be implemented to monitor protocol adherence, identify potential implementation barriers, and resolve operational challenges in a timely manner. This dual-level organizational framework ensures both standardized data collection procedures and individualized participant engagement throughout the study duration.

## Discussion

Global healthcare systems face escalating demands due to population aging, prompting increased focus on interventions to preserve functional capacity and maintain quality of life in older adults through targeted research initiatives and policy reforms. Approximately 1 in 6 community-dwelling older people may have frailty. Data from 120,000 adults aged 60 + from 28 countries show an incidence rate of 43.4 new cases per 1000 person-years [2, 3]. Frailty management has become an increasingly critical focus in geriatric research and clinical practice. Frailty-focused interventions have the potential not only to generate large health care savings but also to lead to important reductions in the physical, emotional, social, and financial problems attributable to disability.

Frailty is characterized by diminished physiological reserve and impaired stress response capacity, clinically manifested by reduced physical activity, sarcopenia, fatigue, and slowed gait velocity. This multisystem dysregulation predisposes to significant functional decline and elevates risks for multiple adverse outcomes including disability, institutionalization, hospitalization, and mortality. Notably, frail older adults demonstrate impaired physical function with heightened vulnerability to rapid functional deterioration and diminished quality of life. Evidence confirms frailty as both an independent predictor of surgical complications, prolonged hospital stays, accelerated deconditioning, and a robust mortality indicator, while concurrently driving substantial healthcare utilization costs [44–49]. 

Frailty is a dynamic reversible state, that can be improved or reversed [11–17]. Compared with disabled elderly people, elderly people with moderate functional limitations are expected to live longer, so improving the functional status of frail elderly people is meaningful. While no definitive gold-standard treatment exists for frailty, current evidence supports multimodal interventions including structured exercise regimens and psychosocial therapies for improving physical function. Optimal management requires a comprehensive, patient-centered approach incorporating systematic identification of individual risk factors.

The advantage of the present study is the method of intervention. The methodological strength of this study lies in its comprehensive intervention approach, which differs from conventional randomized controlled trials targeting single domains (e.g., nutrition, psychology, or physical function). By employing Goal Attainment Scaling (GAS) to establish personalized objectives, we implemented truly individualized interventions tailored to each participant’s specific clinical needs and functional priorities.

GAS is an individualized scale for setting and evaluating progress toward personalized goals, that can be feasible with older adults with multiple chronic conditions and complex needs [34–40]. A study showed that 74% of functionally impaired elderly patients with multiple chronic conditions achieving their designated goals through GAS-based interventions. GAS enhances clinical practice by facilitating patient and caregiver engagement, enabling precise identification of priority concerns, and guiding the implementation of tailored treatment strategies that address each individual’s most salient healthcare requirements.

If a positive consequence could be obtained, a novel treatment for frail elderly patients can be carried out in routine clinical practice. Nonetheless, several study limitations warrant consideration. First, the goal pool was developed through expert consultation, which may introduce selection bias and limit the generalizability of the identified goals. Second, the study was conducted exclusively in Beijing, which possesses substantially greater healthcare resources than most Chinese regions, potentially restricting the applicability of our findings to less resourced settings. Consequently, caution should be exercised when extrapolating these results to other healthcare contexts.

## Supplementary Information

Supplementary Material 1.

## Data Availability

No datasets were generated or analysed during the current study.
