# Supplementary material for: An individualized physical function improvement program for frailty elderly using goal attainment scaling (GAS) as evaluation method: protocol for a randomized controlled trial
Source: BMC Geriatr. 2025 Jul 15;25:528. doi: 10.1186/s12877-025-06203-1 (PMC12261855; doi:10.1186/s12877-025-06203-1)
Supplement: Supplementary file 1 — Supplementary Material 1. [file 12877_2025_6203_MOESM1_ESM.docx]

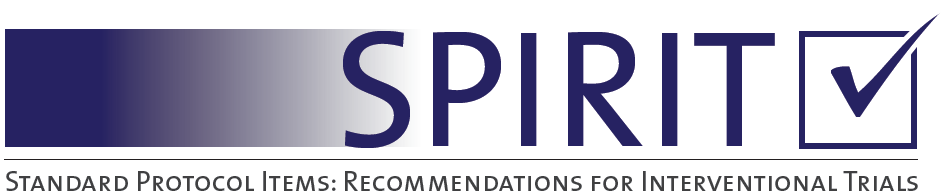


SPIRIT 2013 Checklist: Recommended items to address in a clinical trial protocol and related documents*

| Section/item | ItemNo | Description |
| --- | --- | --- |
| **Administrative information** | | |
| Title | 1 | An Individualized Physical Function Improvement Program for Frailty Elderly Using Goal Attainment Scaling (GAS) as Evaluation Method: protocol for a randomized controlled trial |
| Trial registration | 2a | ClinicalTrials.gov: NCT06919575 |
|  | 2b | **Primary registry and trial identifying numbe**r**:**  ClinicalTrials.gov: NCT06919575  **Date of registration in primary registry:**   1. Apr-02   **Secondary identifying numbers:**  The National Health Commission of the People's Republic of China (2024YB53)  **Sources of monetary or material support:**  The National Health Commission of the People's Republic of China;  Department of Geriatric Medicine, Beijing Chaoyang Hospital  **Sponsor:** Beijing Chao Yang Hospital  **Contact for public queries:**  Name: Xiaojuan Wang, PHD.  Phone Number: +86 13641159814  Email: [xjwang730715@sina.com](mailto:xjwang730715@sina.com?subject=NCT06919575,%20CYYY-Frailty-GAS,%20A%20Individualized%20Physical%20Function%20Improvement%20Program%20for%20Frailty%20Elderly%20Using%20Goal%20Attainment%20Scaling%20(GAS)%20as%20Evaluation%20Method:%20Protocol%20for%20a%20Randomized%20Controlled%20Trial)  **Public title:**  An Individualized Physical Function Improvement Program for Frailty Elderly Using Goal Attainment Scaling (GAS) as Evaluation Method: protocol for a randomized controlled trial  **Scientific title**:  An Individualized Physical Function Improvement Program for Frailty Elderly Using Goal Attainment Scaling (GAS) as Evaluation Method: protocol for a randomized controlled trial  **Countries of recruitment:** China  **Health conditions or problems studied:** Pathologic Processes; Frailty  **Interventions:**  First, the underlying causes of the patient's frailty should be analyzed. Individualized goals should be established using Goal Attainment Scaling (GAS), followed by implementation of tailored measures. For patients presenting with malnutrition or mood-related disorders, comprehensive nutritional and psychological assessments must be conducted, with subsequent nutritional support or pharmacological treatment initiated when clinically indicated.  The patients of the intervention group with poor physical function would receive exercise prescription tailored to the subjects' physical ability from a trained specialist. For participants requiring exercise-based interventions, a moderate and gradually increasing intensity exercise regimen home-base three times a week, over a 12-week duration will be administer under telehealth-based monitoring.  **Key inclusion and exclusion criteria:**  The inclusion criteria were the following:   1. ≥60 years old. 2. Recognized as frailty by Fried scale.   The exclusion criteria were the following:   1. Considered with life expectancy ＜1 year such as advanced cancer patients.   (2) Difficult to communicate with such as patients with severe cognitive impairment (Mini Mental State Examination score ≤ 17).  (3) Severe hearing disorder.  **Study type:**  Primary Purpose: Supportive Care.  Allocation: Randomized.  Interventional Model: Single Group Assignment.  Masking: Triple (Participant, Care Provider, Investigator).  **Date of first enrolment:**  Not yet recruiting.  **Target sample size:** 160.  **Recruitment status:**  Not yet recruiting.  **Primary outcome:**  Goal Attainment Scaling (GAS) formulation of intervention objectives and attainment of goals was assessed using the Goal Attainment Scaling (GAS). GAS is a structured, individualized method for setting and evaluating progress toward personalized goals. Cumulative incidence of any antibiotic associated diarrhoea (time frame: 2 years; not designated as safety issue). The GAS score was calculated according to a standardized formula and assessed at baseline, 3 months, and 24 months.  **Ethics Review:**  The trials have received ethics approval from the Institutional Review Board for Human Studies of Beijing Chaoyang Hospital, Beijing, China.  **Completion Date:** 01-Aug-2027  **IPD Sharing Statement:**  Plan to Share IPD: No |
| Protocol version | 3 | **Issue Date:** 29-May-2025  **Protocol Amendment Number:** 01  **Author:** Xiao-Juan Wang |
| Funding | 4 | This trial is supported by funding from the National Health Commission of the People's Republic of China (Grant No. 2024YB53). We declare that the funding agency has independently reviewed the protocol as part of the process of awarding funding.  In addition to financial support, the Beijing Chaoyang Hospital provides material and logistical support, including access to facilities, clinical staff, medical equipment, and laboratory resources necessary for the implementation of the study.  No commercial funding or industry sponsorship is involved in this study. |
| Roles and responsibilities | 5a | Yu Kang^1*^, Zi-Cheng Qi^1*^, Meng-Tian Guo^1^, Xiao-Juan Wang^1#^  1 Department of Geriatric Medicine, Beijing Chaoyang Hospital, Capital Medical University, Beijing, China  *These authors contributed equally to this work  # Corresponding author: Xiao-Juan Wang (E-mail: xjwangcyyy [@163.com](mailto:shihuanzhong@sina.com))  **Authors’ contributions**  WXJ was involved in the design of the study and undertook funds collection. KY was involved in the design of the study, manuscript preparation, and literature search. QZC contributed to manuscript preparation and literature search. GMT has been involved in the revision of this manuscript. All authors read and approved the final manuscript. |
|  | 5b | **Trial Sponsor:** National Health Commission of the People's Republic of China  **Sponsor’s Reference:** Grant No. 2024YB53  **Contact name:** Xiao-Juan Wang  **Address:** Department of Geriatric Medicine, Beijing Chaoyang Hospital, Capital Medical University, 8 Gongren Tiyuchang Nanlu, Chaoyang District, Beijing 100020, China.  **Telephone:** +86 13641159814  **Email:** xjwangcyyy [@163.com](mailto:shihuanzhong@sina.com) |
|  | 5c | This funding source has no role in the design of this study and will not have any role during its execution, analyses, interpretation of the data, or decision to submit results. |
|  | 5d | **Coordinating Centre**  The trial is coordinated by the Department of Geriatrics Medicine, Beijing Chaoyang Hospital, Capital Medical University, which is responsible for day-to-day trial operations, communication among trial sites, training of investigators, and central logistics. The coordinating team is led by the Principal Investigator (PI), Dr. Xiao-Juan Wang, and includes sub-investigators, a clinical research coordinator, and administrative staff.  **Steering Committee**  A Steering Committee comprising the PI, co-investigators, and subject-matter experts meets regularly to provide scientific oversight, ensure adherence to the protocol, and make high-level decisions on trial conduct. This committee also addresses protocol amendments and operational challenges.  **Data Management Team**  The data management team, based at the coordinating centre, oversees data entry, verification, quality control, and database locking. Double data entry and range checks will be used to ensure data integrity. The team is supervised by an experienced data manager and reports to the PI. |
| Introduction |  |  |
| Background and rationale | 6a | **Background**  Frailty is the deterioration of physiological functions due to aging, linked to negative outcomes. Frailty, being a reversible and dynamic state, calls for individualized and comprehensive management. Goal Attainment Scaling (GAS) is a personalized method for establishing and assessing progress towards individual goals, suitable for older adults with complex requirements. We focus on attaining personalized intervention goals for elderly patients with frailty, correcting frailty manifestations, maintaining enhanced functional capacity, and cultivating sustainable health awareness, with the goal of improving overall quality of life. |
|  | 6b | Routine diagnosis and treatment were chosen as the comparator because they reflect the standard clinical practice currently provided to patients with this condition. This approach ensures that the trial results are directly applicable to real-world settings, enhancing external validity. Comparing the experimental intervention to routine care allows for the evaluation of its additional clinical benefit over existing practices. Moreover, this comparator is ethically justified, as all participants receive at least the recognized standard of care, ensuring patient safety while enabling a pragmatic assessment of the intervention’s effectiveness. |
| Objectives | 7 | **Primary Objective**  To evaluate the efficacy of using Goal Attainment Scaling (GAS) as an evaluation method compared to routine treatment in improving physical function in adults aged 60 years and older with frailty.  **Primary Hypothesis**  Participants receiving an individualized physical function improvement will demonstrate a statistically significant improvement in GAS score compared to those receiving routine care after 3-month and 12-month follow-up.  **Secondary Objectives**  To assess the impact of using GAS as an evaluation method on reversal rate of frailty.To evaluate participant satisfaction and feasibility of the intervention. To explore potential subgroup effects based on age, sex, or baseline severity.  **Secondary Hypotheses**  Using GAS as an evaluation method to set an individualized physical function improvement will reversal rate of frailty compared to routine care. |
| Trial design | 8 | We conducted a randomized clinical trial of an individualized intervention program using GAS as goal setting and evaluation method designed to improve physical functional in frailty elderly. A total of 160 individuals aged ≥60 years, who fulfill the Fried scale of frailty will be recruited from Beijing Chaoyang Hospital, Capital Medical University. All participants set personalized goals through GAS. Patients in the intervention group receive individualized interventions, implement tailored measures based on personalized goals, attain personalized intervention goals to improve or reverse the frailty state. The participants will be followed up for 3 months and 12 months. |
| Methods: Participants, interventions, and outcomes | | |
| Study setting | 9 | We conducted this trial at a large general hospital and two community health service centers within a medical alliance. |
| Eligibility criteria | 10 | **Inclusion criteria**  (1) ≥60 years old.  (2) Recognized as frailty by Fried scale.  **Measures of frailty**  Frailty will be identified using Fried scale according to its five criteria:   1. Weight loss: defined as unintentional weight loss ≥4.5 kilograms or ≥5% of body weight in the last year. 2. Exhaustion: was measured by self-report using two questions: (‘How many days during the last week have you felt that anything you did was a big effort?’ and ‘How many times during the last week have you felt that you could not keep on doing things?’). The criterion was met when participant answered self-reported fatigue ≥3days in the last week. 3. Weakness: based on grip strength, stratified by gender and body mass index (BMI) quartiles. For men, BMI ≤24 kg/m^2^, grip strength ≤29 kg; BMI 24.1–26 kg/m^2^, grip strength ≤30 kg; BMI 26.1–28kg/m^2^, grip strength ≤30 kg; BMI ＞28 kg/m^2^, grip strength ≤32 kg. For women, BMI ≤23 kg/m^2^, grip strength ≤17 kg; BMI 23.1–26 kg/m^2^, grip strength ≤17.3 kg; BMI 26.1–29 kg/m^2^, grip strength ≤18 kg; BMI ＞29 kg/m^2^, grip strength ≤21 kg. In this study, we will measure the grip strength of the dominant hand twice to obtain the average. 4. Slowness: was defined using the 6 m walking test at their usual pace, ≤1.0m/s is considered abnormal. Best time of two performances was chosen.   (5) Low physical activity: defined as energy expenditure of physical activity per week ＜383 kcal for men or ＜270 kcal for women.  The presence of ≥3 criteria is defined as frailty.  **Exclusion criteria**   1. Considered with life expectancy ＜1 year such as advanced cancer patients. 2. Difficult to communicate with such as patients with severe cognitive impairment (Mini Mental State Examination score ≤17).   (3) Severe hearing discord. |
| Interventions | 11a | First, a comprehensive assessment will first identify the underlying etiologies of frailty (e.g., physical inactivity, malnutrition, or psychological factors) to guide targeted interventions. Individualized treatment goals will be established using Goal Attainment Scaling (GAS), with subsequent implementation of personalized intervention plans. For participants presenting with ≥2 concurrent issues, multidisciplinary specialists will coordinate simultaneous management.  Participants identified with malnutrition or mood disorders will undergo standardized nutritional assessment (e.g., Mini Nutritional Assessment) and psychological evaluation (e.g., Geriatric Depression Scale). Evidence-based nutritional supplementation and/or pharmacotherapy will be initiated according to established clinical guidelines when indicated.  The patients of the intervention group with poor physical function would receive exercise prescription tailored to the subjects’ physical ability from a trained specialist. For Participants requiring exercise-based interventions, a moderate and gradually increasing intensity exercise regimen home-based, three times a week will be administer under telehealth-based monitoring. A standardized protocol exercise intervention comprises a 20-30-minute session with progressive phases, over a 12-week duration: 5-minute dynamic stretching warm-up, 10-minute moderate-intensity cardiovascular training, 5-minute progressive resistance exercises, 5-minute proprioceptive neuromuscular facilitation, and 10-minute isometric stance maintenance. Certified exercise specialists will individualize each prescription by modifying intensity, duration, and modality based on comprehensive geriatric assessment, while maintaining core therapeutic components to ensure safety, efficacy, and sustainable adherence. |
|  | 11b | For participants experiencing exercise intolerance, such as dizziness, chest discomfort, or abnormal vital signs during intervention-related physical activity, the session will be stopped immediately. After clinical evaluation, the exercise intensity or duration may be reduced, or the intervention suspended. If the intolerance persists despite adjustment, the participant may be withdrawn from the intervention arm while continuing follow-up under routine care to ensure data completeness and participant safety.  If allergic reactions are suspected withdraw the trial medication from the patient. This should be reported as an adverse event. |
|  | 11c | For behavioural or exercise interventions, participant engagement will be promoted through individualized supervision, goal setting, and progress tracking. Adherence will be monitored through structured exercise logs, attendance records, and, where feasible, wearable activity monitors. Study staff will review adherence data regularly and follow up with participants showing signs of non-compliance.  For pharmacological interventions, participants will be instructed to return all unused medication and empty blister packs at each follow-up visit. Pill counts will be conducted to estimate adherence.  Any barriers to adherence reported by participants will be recorded and addressed through personalized adjustments (e.g., modifying session timing, reducing intensity) to maintain engagement while preserving protocol integrity. |
|  | 11d | During the trial, participants may continue to receive routine medical care for comorbid conditions that are not expected to interfere with the study intervention or outcomes. |
| Outcomes | 12 | The primary outcome measure was Goal Attainment Scaling (GAS) score, which quantitatively assessed individualized goal achievement. The secondary outcome is the reversal rate of frailty, defined as a reversion from frailty to pre-frailty or non-frailty. |
| Participant timeline | 13 | 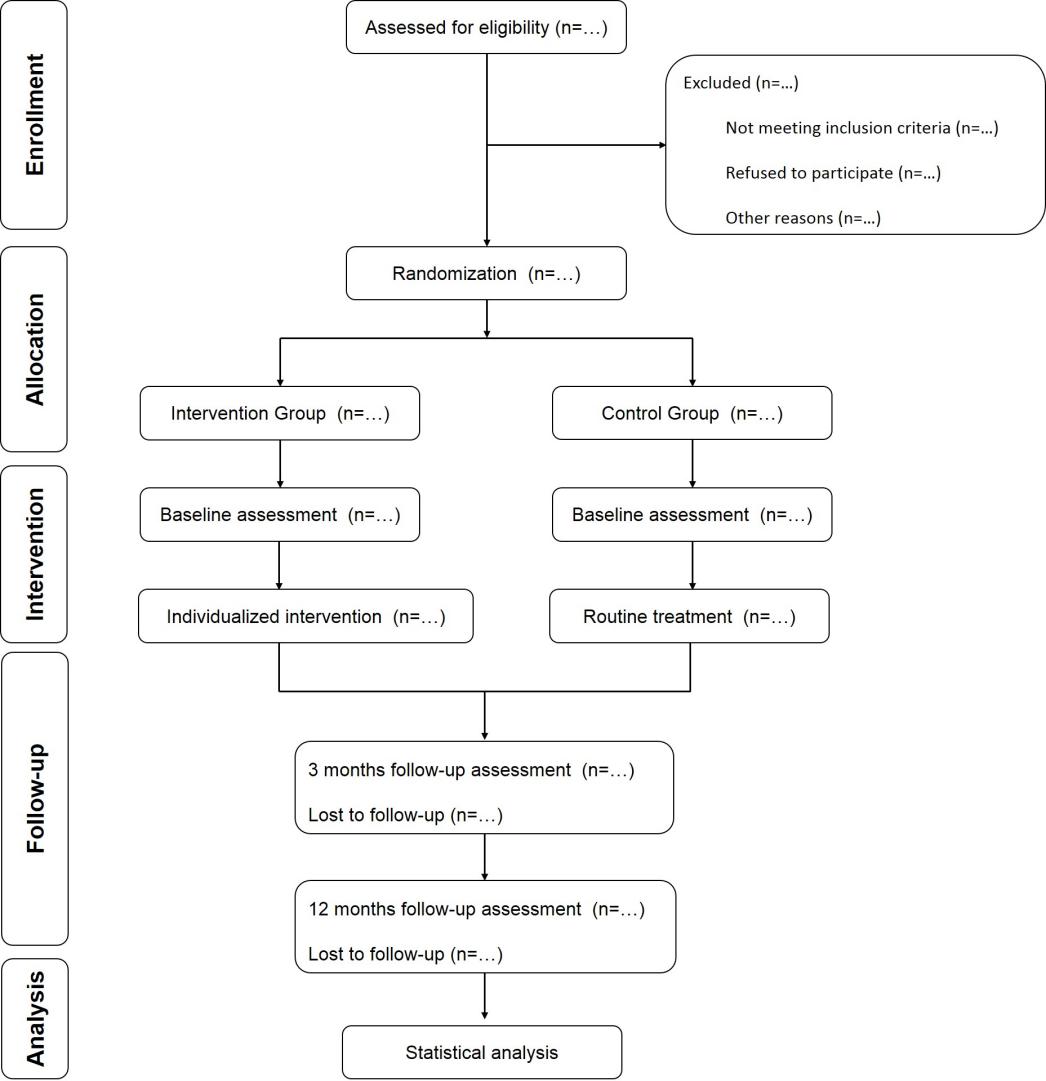 |
| Sample size | 14 | The sample size was calculated using a two-proportion comparison formula for a 1:1 randomized controlled trial, with an α of 0.05, 80% power, and an expected 20% absolute difference in frailty reversal rates between groups, based on prior intervention effect sizes. Initial calculations indicated 64 participants per group. Accounting for a 20% attrition rate, the adjusted sample size was 80 per group, resulting in a total of 160 participants. (<http://powerandsamplesize.com/Calculators/>) |
| Recruitment | 15 | The recruitment process comprises two stages:  **Screening Phase:**  Potential participants aged ≥60 years will be screened for frailty at a large general hospital and two community health service centres within a medical alliance. A brief assessment will evaluate inclusion/exclusion criteria, with outpatient clinics serving as the primary recruitment source.  **Enrolment Phase:**  Eligible individuals expressing interest will receive detailed study information. Written informed consent will be obtained prior to participation. Recruitment will continue until the predetermined sample size for statistical analysis is achieved. |
| **Methods: Assignment of interventions (for controlled trials)** | | |
| Allocation: |  |  |
| Sequence generation | 16a | Once a patient fulfills the criteria, after informed consent, participants will be randomly assigned to either the usual care group or the intervention group based on random number at a 1:1 ratio generated in a blinded manner by the computerized randomization system. |
| Allocation concealment mechanism | 16b | The information of random group allocation will be maintained in a sealed envelope opened only by a trial manager who is not involved in the recruitment, assessment, or intervention to assign the treatment. |
| Implementation | 16c | The allocation sequence will be generated by an independent statistician using a computer-generated randomization program. The randomization list will be securely stored and concealed from the study investigators involved in participant enrolment and assessment.  Participant enrolment will be conducted by investigators at each site, based on pre-specified inclusion and exclusion criteria.  After a participant is deemed eligible and has provided written informed consent, the assignment to a study group will be performed by a designated study staff member who is not involved in outcome assessment. Allocation will be implemented via a centralized randomization system (sealed opaque envelopes) to ensure allocation concealment. |
| Blinding (masking) | 17a | In this trial, the outcome assessors and data analysts will be blinded to group allocation to minimize detection and analysis bias.  Trial participants and care providers will also be blinded. All efforts will be made to ensure that participants are unaware of the study hypotheses and expected effects to reduce expectation bias.  To maintain blinding of outcome assessors, separate personnel will be assigned for data collection and intervention delivery. Assessors will not have access to the randomization list or any documentation revealing group allocation. Data analysts will receive anonymized datasets labeled only with group codes, with the allocation key withheld until primary analysis is complete. |
|  | 17b | In this trial, unblinding will be limited to cases where knowledge of the participant’s intervention allocation is necessary to ensure their safety or guide emergency clinical management (e.g., in the case of a serious adverse event potentially related to the intervention).  If unblinding is required, the decision must be made by the principal investigator and documented with justification. Only designated clinical staff (not involved in outcome assessment) will be permitted to access the allocation information through a secure, access-controlled system.Outcome assessors and data analysts will remain blinded throughout the study.  Participants who are unblinded may continue in the study for safety monitoring and data collection unless otherwise deemed necessary to withdraw. |
| **Methods: Data collection, management, and analysis** | | |
| Data collection methods | 18a | **Primary outcome**  The GAS process involves: (1) goal identification; (2) baseline status determination; (3) specification of outcome levels on a five-point ordinal scale (-2 to +2); (4) goal weighting; and (5) follow-up scoring by comparing achieved outcomes with predefined attainment levels. This structured approach enables quantitative measurement of intervention efficacy across diverse clinical targets.  Goal Attainment Scaling (GAS) scores were calculated using a standardized formula and evaluated at baseline, 3-month, and 12-month follow-ups. An aggregated T score with a mean of 50 and an SD of 10 was obtained. T is the composite score, wi is the weight assigned to the ith goal, xi is the numerical value (− 2 to +2) of the attainment level of the ith goal. The extent to which goals are achieved is standardised into a T score by the formula: $GAS score= 50 + \frac{[10\sum\left( \mathrm{wi}\times\mathrm{xi} \right)]}{\surd[0.7\sum{wi}^{2}+{0.3\left( \sum wi \right)}^{2}]}$  **Secondary outcome**  The secondary outcome is the reversal rate of frailty, defined as a reversion from frailty to pre-frailty or non-frailty.  The study will collect demographic and baseline functional information from the patient’s legally authorized representative and/or caregivers. |
|  | 18b | To maximize participant retention and ensure completeness of follow-up, several strategies will be employed:  Regular contact through phone calls, text messages, or digital communication platforms to remind participants of upcoming visits and reinforce engagement.  Flexible scheduling of follow-up appointments to accommodate participants’ availability.  Clear communication of the importance of continued participation, even if intervention adherence is suboptimal.  Provision of small, ethically approved incentives when appropriate.  For participants who discontinue the assigned intervention or deviate from the protocol, efforts will be made to collect primary and secondary outcome data at all planned follow-up time points, unless the participant explicitly withdraws consent for further data collection. |
| Data management | 19 | All trial data will be entered into a secure, password-protected electronic data capture system designed with built-in logic and range checks to minimize entry errors. Data will be entered by trained personnel at each site and monitored regularly for completeness and accuracy.  Double data entry will be performed for critical variables to ensure data accuracy. Automated range and consistency checks will be implemented in real-time to detect outliers, missing values, and inconsistencies. Queries will be issued and resolved through the system before data locking.  Regular data backups will be scheduled, and a complete audit trail of data modifications will be maintained.  Details of data management procedures, including the data management plan, are described in a separate document and will be available upon request or accessible via the trial’s internal documentation platform. |
| Statistical methods | 20a | The intervention group will be compared against the control group for all primary analysis. We will use chi-squared test for binary outcomes, and *t*-test for continuous outcomes. For subgroup analyses, we will use regression methods with appropriate interaction terms.  Multivariable analyses will be based on logistic regression for binary outcomes and linear regression for continuous outcomes. We will examine the residual to assess model assumptions and goodness-of-fit. *P*-values will be reported to four decimal places with *p*-values less than 0.001 reported as *p* < 0.001. For all tests, we will use 2-sided *p*-values with α ≤ 0.05 level of significance. We will use the Bonferroni method to appropriately adjust the overall level of significance for multiple primary outcomes, and secondary outcomes. |
|  | 20b | **Subgroup Analyses:**  Pre-specified subgroup analyses will be conducted to explore potential effect modification. The subgroups include (but are not limited to):  Age groups  Sex  Baseline severity of condition  Comorbidities  Interaction terms between intervention group and subgroup variables will be included in regression models to assess heterogeneity of intervention effects.  **Adjusted Analyses:**  Multivariable regression models will be used to adjust for potential baseline imbalances and known confounders, such as age, sex, and other clinically relevant covariates. |
|  | 20c | We propose to test non-inferiority using two analysis sets; the intention-to-treat set, considering all patients as randomized regardless of whether they received the randomized treatment, and the “per protocol” analysis set. Given our expectation that very few patients will crossover or be lost to follow-up, these analyses should agree very closely. |
| **Methods: Monitoring** | | |
| Data monitoring | 21a | An independent Data Monitoring Committee (DMC) will be established to periodically review trial conduct, recruitment progress, data quality, participant safety, and interim outcome data. The DMC will consist of at least three members, including:  One senior clinical expert in the study field.  One biostatistician with experience in clinical trial methodology.  One member with ethical or regulatory expertise.  All members will be independent from the sponsor, the trial steering committee, and the study investigators. They will not have any financial or competing interests related to the trial.  The DMC will meet at predefined intervals, and will make recommendations regarding trial continuation, modification, or early termination. Their recommendations will be reported to the principal investigator and the trial sponsor in a timely and confidential manner. |
|  | 21b | **Interim Analyses:**  One interim analysis is planned after [e.g., 50%] of the total target sample has completed the primary outcome assessment. The interim analysis will be conducted primarily to evaluate:  Participant safety.  Recruitment and retention progress.  Early evidence of benefit or harm.  **Stopping Guidelines:**  Pre-specified stopping criteria will be based on:   1. Evidence of significant harm to participants in either group. 2. Overwhelming evidence of benefit (using conservative thresholds such as *p*<0.001 to avoid type I error inflation). 3. Futilit.   **Access to Interim Results and Decision-Making:**  Interim results will be reviewed only by the DMC. The trial investigators, care providers, and sponsor will remain blinded to interim results unless a formal recommendation is made to modify or terminate the trial.  The final decision to terminate or continue the trial will be made jointly by the principal investigator and the sponsor, based on the DMC’s recommendation |
| Harms | 22 | **Collection of Adverse Events (AEs):**  All solicited and spontaneously reported adverse events (AEs), and serious adverse events (SAEs) will be actively monitored and documented throughout the study period, from the time of enrolment until final follow-up visit.  Participants will be queried at each study visit regarding any adverse symptoms or events, and research staff will also record any clinical signs or abnormal test results.  **Assessment of AEs:**  Each AE will be evaluated for:  Severity  Causality  Expectedness  Assessment will be conducted by qualified study physicians or investigators.  **Reporting Procedures:**  All SAEs will be reported to the trial sponsor and ethics committee within 24–72 hours of becoming known.AEs will be summarized in regular progress reports and the final trial report.  **Management of AEs:**  Participants experiencing AEs will receive appropriate clinical care.  Any intervention may be modified or discontinued at the discretion of the investigator based on participant safety.  All safety decisions will be documented in the case report form and source records. |
| Auditing | 23 | **Auditing Procedures and Frequency:**  Independent audits of the trial conduct will be carried out to ensure compliance with the protocol, Good Clinical Practice (GCP), and applicable regulatory requirements.  Audits will be conducted at predefined milestones by a qualified, independent auditor who is not part of the sponsor organization or trial investigation team. The auditor will review:  Participant consent processes.  Data accuracy and completeness.  Adverse event documentation and reporting.  Protocol adherence.  The findings will be documented in formal audit reports, which will be shared with the sponsor and principal investigator. Any major findings or deviations will be followed up with corrective actions and re-audits as needed. |
| Ethics and dissemination | | |
| Research ethics approval | 24 | The trials have received ethics approval from the Institutional Review Board for Human Studies of Beijing Chaoyang Hospital, Beijing, China. Any subsequent protocol amendments will also be submitted to the Institutional Review Board for review and approval prior to implementation. |
| Protocol amendments | 25 | Any modifications to the protocol which may impact on the conduct of the study, potential benefit of the patient or may affect patient safety, including changes of study objectives, study design, patient population, sample sizes, study procedures, or significant administrative aspects will require a formal amendment to the protocol. Such amendment will be approved by the Institutional Review Board for Human Studies of Beijing Chaoyang Hospital, Beijing, China prior to implementation and notified to the health authorities in accordance with local regulations. |
| Consent or assent | 26a | Informed consent will be obtained by trained inpatient physicians who are members of the study team. Prior to any study-related procedures, each potential participant aged 60 years or older will be approached in a private and quiet setting within the hospital ward.  The purpose, procedures, risks, and benefits of the study will be explained using an ethics committee–approved participant information sheet, written in language suitable for older adults.  Participants will be given adequate time to consider participation and may consult with family members or caregivers if they wish. Written informed consent will be obtained in duplicate: one copy will be kept in the study file, and one will be provided to the participant. |
|  | 26b | No additional consent will be sought for the collection or future use of biological specimens, as this study does not involve the collection of any biological samples for ancillary research purposes. |
| Confidentiality | 27 | Personal information about potential and enrolled participants will be collected and managed in accordance with applicable data protection laws and institutional policies.  All participants will be assigned a unique study identification code, and identifiable information will be stored separately from research data in a secure, access-restricted database.  During data collection and analysis, only coded (de-identified) data will be used. Access to identifiable information will be limited to authorized study staff responsible for enrolment, follow-up, or safety monitoring.  No personal identifiers will appear in publications or reports. Data will be retained for 5 years following study completion, after which it will be securely destroyed or archived in accordance with institutional policy. |
| Declaration of interests | 28 | The members of the study team declare that they have no competing interests. |
| Access to data | 29 | Only authorized members of the study team will have access to the final, cleaned trial dataset. Specifically, access will be granted to the principal investigator, designated co-investigators, and the trial statistician.  The data will be stored on a secure, password-protected server with role-based access controls.  There are no contractual agreements that restrict the investigators’ access to the full dataset. The study sponsor does not impose any limitations on data access, analysis, or publication decisions made by the research team.  Any requests for data sharing outside the study team will be subject to ethical approval and de-identification of participant information. |
| Ancillary and post-trial care | 30 | Participants will continue to receive standard medical care throughout the trial and following its completion. The trial does not involve withholding any necessary or proven effective treatment.  No specific ancillary or post-trial care is planned beyond the scheduled follow-up, as the intervention is of low risk and does not involve long-term dependency or novel medications.  In the unlikely event that participant experiences harm directly related to trial participation, compensation and medical treatment will be provided in accordance with institutional policies and national regulations. |
| Dissemination policy | 31a | The results of this trial will be disseminated to relevant stakeholders through multiple channels, including:  Peer-reviewed journal publications.  Presentations at national and international scientific conferences.  Submission to clinical trial registries.  Lay summaries for participants.  The research team is committed to transparent and timely reporting of trial findings, regardless of study outcomes.  There are no contractual publication restrictions imposed by the sponsor. All publications and presentations will be prepared by the investigators and submitted in accordance with authorship criteria and institutional guidelines. |
|  | 31b | Authorship of all trial-related publications will be determined in accordance with the International Committee of Medical Journal Editors (ICMJE) guidelines.  Individuals will be eligible for authorship if they meet all the following criteria:   1. Substantial contributions to the conception or design of the work; or the acquisition, analysis, or interpretation of data. 2. Drafting the work or revising it critically for important intellectual content. 3. Final approval of the version to be published. 4. Agreement to be accountable for all aspects of the work.   Contributors who do not meet all four criteria will be acknowledged in the publication’s Acknowledgements section.  No professional medical writers will be used in preparing the trial manuscripts. All writing will be conducted by the investigators and co-authors involved in the study. |
|  | 31c | The full trial protocol will be made publicly available as a supplementary file alongside the main publication of trial results or uploaded to ClinicalTrials.gov (ChiCTR) as required.  De-identified participant-level datasets and the statistical analysis code may be shared upon reasonable request for academic research purposes, subject to approval by the principal investigator and institutional ethics committee.  All shared data will be de-identified to protect participant confidentiality, and data requestors will be required to sign a data use agreement outlining appropriate use, data protection measures, and publication responsibilities. |
| Appendices |  |  |
| Informed consent materials | 32 | A model participant information sheet and informed consent form (ICF) has been developed in accordance with institutional ethics guidelines and national regulations.  These documents clearly explain the study’s purpose, procedures, risks and benefits, data confidentiality, voluntary participation, and contact information for questions or concerns.  The consent form will be provided in language that is easily understandable to the target population in simplified Chinese, and consent will be obtained prior to any trial procedures.  Copies of the model consent form and related documentation are available upon request or will be submitted as part of the ethics committee application package. |
| Biological specimens | 33 | No biological specimens will be collected from participants as part of this trial.Therefore, there are no plans for the laboratory evaluation or storage of specimens for genetic or molecular analysis, either for the current trial or for future ancillary studies. |

*It is strongly recommended that this checklist be read in conjunction with the SPIRIT 2013 Explanation & Elaboration for important clarification on the items. Amendments to the protocol should be tracked and dated. The SPIRIT checklist is copyrighted by the SPIRIT Group under the Creative Commons “[Attribution-NonCommercial-NoDerivs 3.0 Unported](http://www.creativecommons.org/licenses/by-nc-nd/3.0/)” license.
